# Supplementary material for: Unravelling Effects of Rosemary (Rosmarinus officinalis L.) Extract on Hepatic Fat Accumulation and Plasma Lipid Profile in Rats Fed a High-Fat Western-Style Diet
Source: Metabolites. 2023 Aug 27;13(9):974. doi: 10.3390/metabo13090974 (PMC10534343; doi:10.3390/metabo13090974)
Supplement: Supplementary file 1 [file metabolites-13-00974-s001.zip › metabolites-2524718-supplementary.pdf]

Unravelling Effects of Rosemary (*Rosmarinus officinalis* L.) Extract on Hepatic Fat Accumulation and Plasma Lipid Profile in Rats Fed a High-Fat Western-Style Diet

Sidsel Madsen [1], Steffen Yde Bak [2], Christian Clement Yde [2], Henrik Max Jensen [2], Tine Ahrendt Knudsen [2], Cecilie Bæch-Laursen [3], Jens Juul Holst [3], Christoffer Laustsen [4], Mette Skou Hedemann[1,*]

[1] Department of Animal and Veterinary Sciences, Aarhus University, Blichers Allé 20, DK-8830 Tjele, Denmark

[2] IFF—Nutrition Biosciences Aps, Edwin Rahrs Vej 38, DK-8220 Brabrand, Denmark

[3] Department of Biomedical Sciences and Novo Nordisk Foundation, Center for Basic Metabolic Research, Faculty of Health and Medical Sciences, University of Copenhagen, Blegdamsvej 3, DK-2200 Copenhagen, Denmark

[4] The MR Research Centre, Department of Clinical Medicine, Aarhus University, Palle Juul-Jensens Boulevard 99, DK-8200 Aarhus, Denmark

* Correspondence: mette.hedemann@anivet.au.dk; Tel.: +45-51448783

Supplementary methods

Food intake measurements

Twice a week the feeding cups were cleaned and approximately 75 g feed (exact weight was noted) was weighed out for each cage. The leftover was weighed 24h later and feed intake was calculated by subtracting the leftover feed from the amount given and divided by the number of rats in the cage (two). The rats were weighed individually twice a week, on the day the weighed-out feed was provided.

Studies on isolated perfused rat small intestine

On the day of experiment, rats were anesthetized with a subcutaneous injection of hypnorm/midazolam (0.3 mL/100 g body weight, per ml: 0.08 mg fentanyl, 2.5 mg fluanisone, 0.45 mg Methyl Parahydroxybenzoate, 0.05 mg Propyl Parahydroxybenzoate, Midazolam: 1.25 mg, Matrix Pharmaceuticals, Hellerup, Denmark) to block the activity of pain sensing nerves and to induce surgical anesthesia. The rat was placed on a heated operating table (37°C), the abdominal cavity was opened by a mid-line incision and the large intestine was excised after tying off the supplying vasculature. The entire small intestine (≈ 100 cm) was perfused in situ by insertion of a catheter in the upper mesenteric artery and perfusion effluent was collected through a catheter inserted in the portal vein. The perfusion buffer consisted of a Krebs-Ringer bicarbonate buffer supplemented with 0.1% (w/v) BSA (fraction V), 5% (w/v) dextran T-70 (to balance oncotic pressure [Pharmacosmos, Holbaek, Denmark]), and 3.5 mmol/L glucose, and 5 mmol/L pyruvate, fumarate, and glutamate. pH was adjusted to 7.4-7.5). A flow rate of 7.5 mL/min was used. Prior to perfusion, the perfusion buffer was maximally gassed with 95% O2 and 5% CO2 to maximize oxygen partial pressure and perfusion buffer was passed through a UP100 Universal Perfusion System from Hugo Sachs (Harvard Apparatus, March Hugstetten, Germany), which includes heating to 37°C. Test stimulus consisted of RE (20 µg/mL) which was administered intra-arterially and intra-luminally to the perfused gut in the mentioned order. Test compound was prepared in isotonic saline for luminal stimulation and in perfusion buffer for intra-arterial stimulation. Luminal stimulation was initiated with a bolus administration at a rate of 2.0 mL/min for the first five min (to quickly expose most of intestinal lumen to the test compound) followed by a flow rate of 0.5 mL/min for the remaining of the stimulation period. Immediately after the stimulation period, the lumen was flushed with isotonic saline at a flow rate of 2.0 mL/min for the first five minutes and then at 0.5 mL/min for the remaining of the experiment. Saline solutions were in all cases room

temperature. At the end of experiment, intra-arterial taurodeoxycholic acid (TDCA), a well-known GLP-1 secretagogue, was infused intra-arterial to control for responsiveness. TDCA stimulated a robust and instant secretory response in all experiments.

SCFA analysis

In brief, 100 µg of intestinal sample was diluted 10-fold with a 0.028 M NaOH solution containing internal standard (2-ethylbutyric acid, Aldrich, Brøndby, Denmark) and homogenised for two minutes. 125 µL 37 % HCl and 500 µL diethyl ether were added, and the sample was vortex for 30 s, before centrifuged (5,000 *x* g, 5°C for 5 min). Next, 50 µL of the ether layer was transferred to a vial and 10 µL of derivatisation reagent N-methyl –N-t-butyldimethylsilyl trifluoroacetamide (Honeywell Fluka, NJ, USA) was added. The reaction mixture was briefly vortex and incubated at 80°C for 20 min, followed by a further incubation at room temperature for 48 hours. Quantification of SCFA, was conducted on a Thermo Scientific™ TRACE™ 1310 Gas Chromatograph equipped with a flame ionisation detector and a 30 m Phenomenex Zebron ZB-1 column with an internal diameter of 0.25 mm coated with 100 %-dimethylpolysiloxane with a film thickness of 0.25 µm. The samples were injected with a Thermo Scientific™ autoinjector (Model AI 1310) Injector temperatures were set to 250°C and detector temperatures were set to 300°C. The carrier gas was helium with a constant pressure of 17.00 psi and makeup gas flow was 40 mL/min of helium. A sample volume of 2 µL was injected with a split flow of 30 mL/min and 3.5 mL/min of purge flow. The compounds were eluted with a temperature gradient of the following shape: held at 70°C for 1 min then increased to 110°C (4°C per min), then increased to 180°C (15°C per min), further increased to 310°C (25°C per min) and held for 3.13 min. The chromatograms were integrated using Thermo Scientific™ Dionex™ Chromeleon™ 7 chromatography software.

Supplementary results

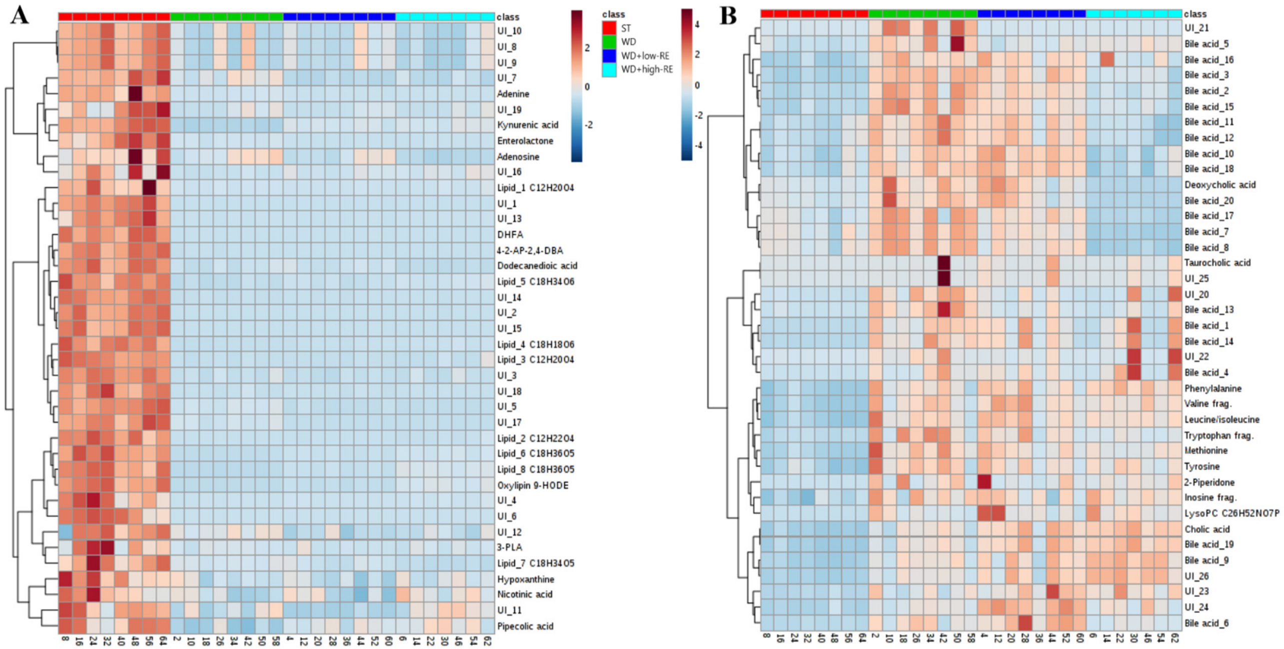

Figure S1: Heat maps of metabolites discriminating between the ST and the WD diet identified and annotated in cecal content of rats and being dominant in the (A) ST diet or (B) WD diet, respectively. Abbreviations: UI, Unidentified; DHFA, Dihydroferulic acid; 4-(2-AP)-2,4-DBA, 4-(2-Aminophenyl)-2,4-dioxobutanoic acid, 3-PLA, 3-Phenyllactic acid; Frag., Fragment; ST, standard diet; WD, Western style diet; WD+low-RE, WD supplemented with low concentration of rosemary extract; WD+high-RE, WD supplemented with high concentration of rosemary extract.

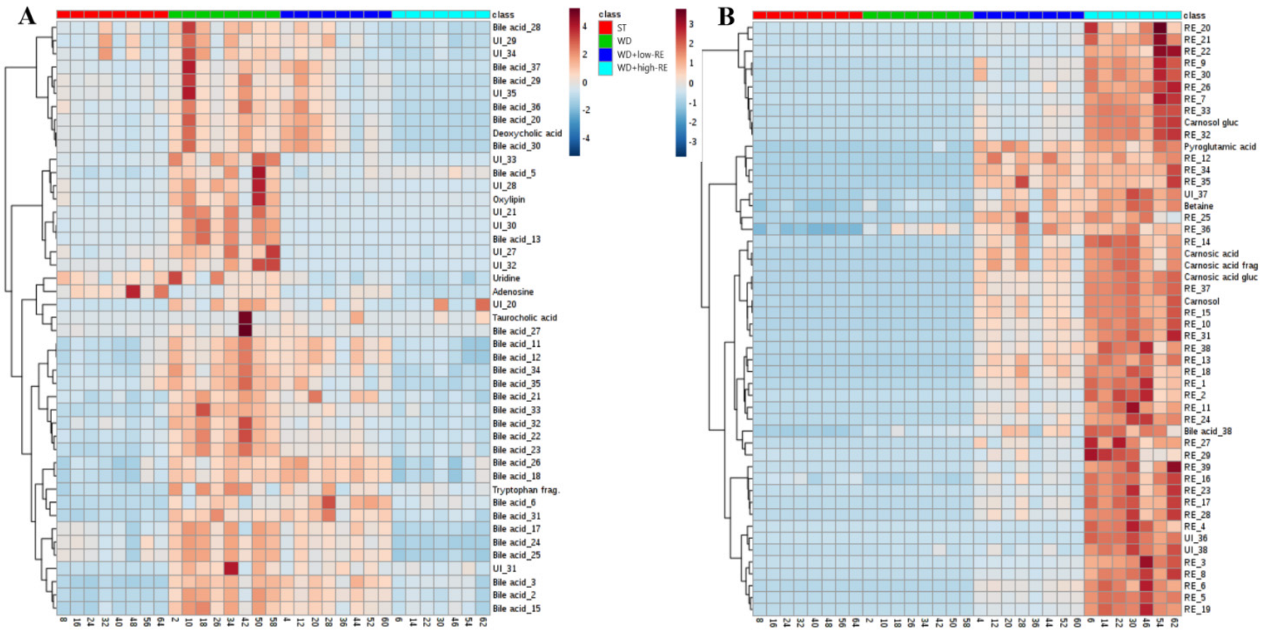

Figure S2: Heat maps of metabolites discriminating between the WD and the WD+high-RE diet identified and annotated in caecal content of rats and being dominant in the (A) WD diet or (B) WD+high-RE diet, respectively. Abbreviations: UI, Unidentified; frag., fragment; RE, Metabolite derived from rosemary extract; gluc, glucuronide; ST, standard diet; WD, Western style diet; WD+low-RE, WD supplemented with low concentration of rosemary extract; WD+high-RE, WD supplemented with high concentration of rosemary extract.

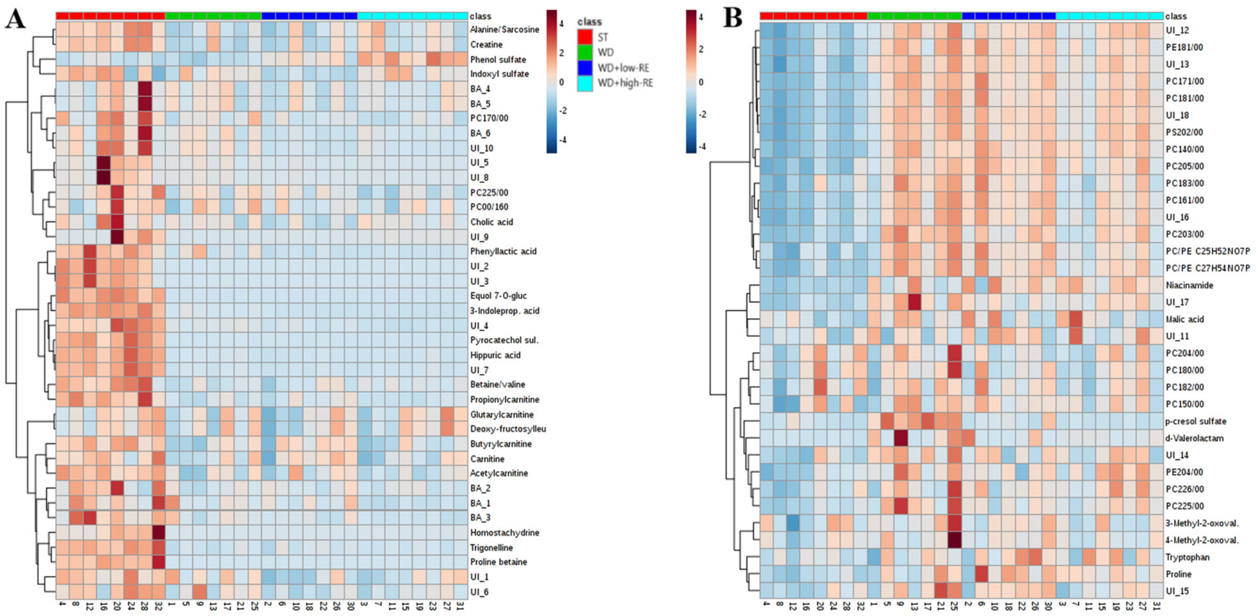

Figure S3: Heat maps of metabolites discriminating between the ST and the WD diet identified and annotated in plasma of rats and being dominant in the a) ST diet or b) WD diet, respectively. Abbreviations: BA, Bile acid; UI, Unidentified; Equol 7-O-gluc, Equol 7-O-glucuronide; 3-indoleprop. acid, 3-Indolepropionic acid; Pyrocatechol sul., Pyrocatechol sulfate; Deoxy-fructosylleu, N-(1-Deoxy-1-fructosyl)leucine; 3-Methyl-2-oxoval., 3-Methyl-2-oxovaleric acid; 4-Methyl-2-oxoval., 4-Methyl-2-oxovaleric acid; ST, standard diet; WD, Western style diet; WD+low-RE, WD supplemented with low concentration of rosemary extract; WD+high-RE, WD supplemented with high concentration of rosemary extract.

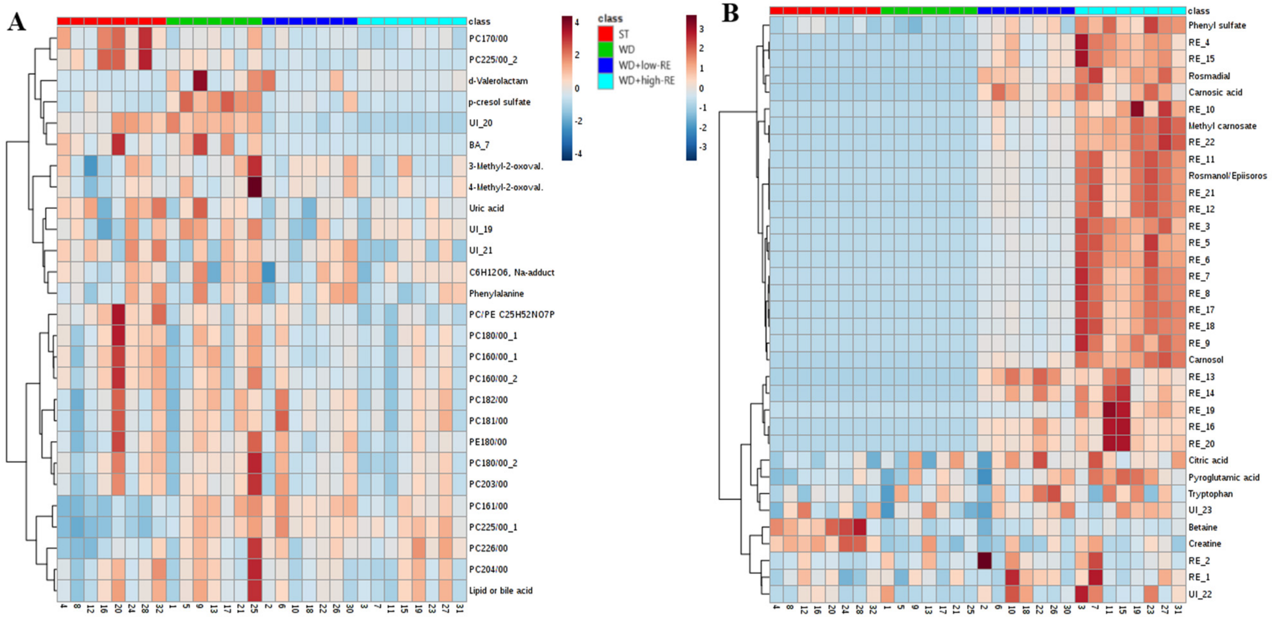

Figure S4: Heat maps of metabolites discriminating between the WD and the WD+high-RE diet identified and annotated in plasma of rats and being dominant in the a) WD diet or b) WD+high-RE diet, respectively. Abbreviations: UI, Unidentified; 3-Methyl-2-oxoval., 3-Methyl-2-oxovaleric acid; 4-Methyl-2-oxoval., 4-Methyl-2-oxovaleric acid; RE, Metabolite derived from rosemary extract; Rosmanol/Epiisoros, Rosmanol/Epiisorosmanol. Class: 1, standard diet (ST); 2, Western style diet (WD), or WD supplemented with either 3, low (low-RE) or 4, high (high-RE) concentrations of rosemary extract.

Supplementary Table S1. Ingredient composition and chemical composition[1] of standard diet (ST), Western style diet (WD), or a WD supplemented with either low (low-RE) or high (high-RE) concentrations of rosemary extract.

	ST[2]	WD	WD+low-RE	WD+high-RE
	g/1000 g			
Casein		194.7	194.5	193.3
DL-Methionine		3.0	3.0	3.0
Corn starch		49.9	49.9	49.7
Maltodextrin 10		99.8	99.7	99.4
Sucrose		340.5	340.1	339.1
Cellulose		49.9	49.9	49.7
Mink fat, anhydrous		199.7	199.5	198.9
Corn oil		10.0	10.0	9.9
Mineral mix[3]		34.9	34.9	34.8
Calcium carbonate		4.0	4.0	4.0
Vitamin mix[4]		10.0	10.0	9.9
Choline bitartrate		2.0	2.0	2.0
Cholesterol		1.5	1.5	1.5
Ethoxyquin		0.04	0.04	0.04
Rosemary extract		0	1.0	4.0
	Chemical composition, g/100 g dry matter			
Crude protein (N x 6.25)	23.9	19.2	18.9	18.7
Crude fat	4.71	20.09	20.26	21.93
Ash	5.47	3.14	3.19	3.05
Starch	31.3	15.0	14.9	15.2

Digestible carbohydrates (calculated[5])	44.3	51.6	51.0	50.6
Non-starch polysaccharides	18.8	4.6	4.4	4.8
Dietary fibre	21.5	6.0	6.6	5.7
	Relative energy contribution (%)[6]			
Carbohydrates	50.0	43.9	43.5	42.3
Fat	11.6	37.3	37.6	39.9
Protein	27.0	16.4	16.1	15.6
Dietary fibre	11.4	2.4	2.7	2.2

[1]Diets were analysed as previously described.

[2]Altromin 1324, Maintenance diet for rats and mice (www.altromin.com).

[3]The mineral mix provided the following quantities of minerals per kilogram of complete diet: 15.4 g of potassium citrate, monohydrate, 5.2 g of NaCl, 3.6 g of potassium sulfate, 1.7 g Mg as MgO, 0.42 g Fe as ferric citrate, 0.24 g Mn as $Co_3Mn \cdot H_2O$, 0.11 g Zn as $ZnCo_3$, 0.04 g Cr as chromium potassium sulfate, 0.02 g Cu as $CuCO_3$, 0.0007 g I as KIO_3, 0.007 g Se as $NaSeO_3$.

[4]The vitamin mix provided the following quantities of minerals per kilogram of complete diet: 1.0 g Vitamin E acetate (50%), 0.3 g niacin, 0.2 g biotin (1%), 0.16 g pantothenic acid, 0.1 g vitamin D3 (100.000 IU/g), 0.1 g vitamin B12, 0.08 g vitamin A acetate (500.000 IU/g), 0.07 g pyridoxine, 0.06 g riboflavin, 0.06 g thiamine, 0.02 g folic acid, 0.008 g menadione sodium bisulfate.

[5]Calculated as (100 – crude protein – crude fat – ash – dietary fibre).

[6]Calculated using the FAO energy conversion factors for carbohydrates (17 kJ/g), protein (17 kJ/g), fat (37 kJ/g), and dietary fibre (8 kJ/g).

Supplementary Table S2. Gene and protein name of quantified proteins involved in metabolic detoxification pathways. Fold changes between the different diets in rats fed standard diet (ST), or a WD supplemented with either low (low-RE) or high (high-RE) concentrations of rosemary extract, measured at day 46. n=8

Protein name	Gene	WD/ST	WD+low-RE/ST	WD+high-RE/ST
Cytochrome P450 1A2	CYP1A2	0.5	0.4	0.3
Cytochrome P450 3A2	CYP3A2	1.1	0.8	0.9
Cytochrome P450 4A2	Cyp4a2	1.5	1.1	1.5
UDP-glucuronosyltransferase 2B1	UGT2B1	1.29	1.25	1.29
Glutathione S-transferase Mu 2	GSTM2	0.80	0.98	1.06
Liver carboxylesterase 4		1.03	1.16	1.40
UDP-glucuronosyltransferase 2B37	UGT2B37	0.96	1.00	1.37
UDP-glucuronosyltransferase 1-1	UGT1A1	1.11	1.21	1.59
Glutathione S-transferase Yb-3	GSTM3	0.84	1.10	1.21
Glutathione S-transferase Mu 1	GSTM1	0.95	1.32	1.38
UDP-glucuronosyltransferase 2B15	UGT2B15	0.74	0.88	1.13
Glutathione S-transferase alpha-5	GSTA5	0.81	0.96	1.24
Glutathione reductase (Fragment)	GSR	0.99	1.22	1.66
UDP-glucuronosyltransferase 1-5	UGT1A5	1.06	1.35	1.82
Glutathione S-transferase theta-1	GSTT1	1.17	1.53	2.30
UDP-glucuronosyltransferase 1-6	UGT1A6	1.05	1.52	2.26
Aflatoxin B1 aldehyde reductase member 3	AKR7A3	0.92	3.03	5.47

Supplementary Table S3. List of metabolic features discriminating between rats fed ST and WD in caecal content

Diet	Ionization mode	m/z[1]	RT[2]	Ion	Metabolite	KEGG/HMDB[3]	Pathway	ID Level
ST	NEG	135.0313	1.01	$[M-H]^-$	Hypoxanthine	C00262	Purine metabolism	1
ST	NEG	188.0354	3.13	$[M-H]^-$	Kynurenic acid	C01717	Tryptophan metabolism	1
ST	NEG	206.0459	3.42	$[M-H]^-$	4-(2-Aminophenyl)-2,4-dioxobutanoic acid	C01252	Tryptophan metabolism	2
ST	NEG	285.1707	3.63	Unknown	UI_1[4]			4
ST	NEG	227.1291	3.68	$[M-H]^-$	Lipid_1 ($C_{12}H_{20}O_4$)[5]		Fatty acids and conjugates metabolism	3
ST	NEG	195.0664	3.78	$[M-H]^-$	Dihydroferulic acid	HMDB0062121	Potential serum biomarker of whole grain intake	2
ST	NEG	229.1446	3.99	$[M-H]^-$	Lipid_2 ($C_{12}H_{22}O_4$)		Fatty acids and conjugates metabolism	2
ST	NEG	190.0510	4.03	$[M-H]^-$	UI_2			4
ST	NEG	227.1290	4.16	$[M-H]^-$	Lipid_3 ($C_{12}H_{20}O_4$)		Fatty acids and conjugates metabolism	3
ST	NEG	361.2232	4.34	$[M-H]^-$	UI_3			4
ST	NEG	165.0559	4.47	$[M-H]^-$	3-Phenyllactic acid	C05607	Phenylalanine metabolism	1
ST	NEG	337.0387	4.56	Unknown	UI_4			4
ST	NEG	315.1239	4.85	Unknown	UI_5			2
ST	NEG	329.1032	5.09	$[M-H]^-$	Lipid_4 ($C_{18}H_{18}O_6$)		Fatty acids and conjugates metabolism	3
ST	NEG	273.0768	6.07	Unknown	UI_6			4
ST	NEG	345.2282	6.33	$[M-H]^-$	Lipid_5 ($C_{18}H_{34}O_6$)		Fatty acids and conjugates metabolism	3
ST	NEG	331.2489	6.42	$[M-H]^-$	Lipid_6 ($C_{18}H_{36}O_5$)		Fatty acids and conjugates metabolism	3
ST	NEG	329.2333	6.54	$[M-H]^-$	Lipid_7 ($C_{18}H_{34}O_5$)		Fatty acids and conjugates metabolism	3
ST	NEG	297.1132	6.62	$[M-H]^-$	Enterolactone	C18165		1
ST	NEG	229.1445	6.81	$[M-H]^-$	Dodecanedioic acid	C02678	Fatty acids and conjugates metabolism	2
ST	NEG	331.2489	6.89	$[M-H]^-$	Lipid_8 ($C_{18}H_{36}O_5$)		Fatty acids and conjugates metabolism	3
ST	NEG	359.1899	7.38	Unknown	UI_7			4

ST	POS	71.0293	0.57	Unknown	UI_8			4
ST	POS	82.5373	0.57	Unknown	UI_9			4
ST	POS	128.0195	0.57	Unknown	UI_10			4
ST	POS	136.0621	0.81	$[M+H]^+$	Adenine	C00147	Purine metabolism	1
ST	POS	160.1336	0.83	Unknown	UI_11			4
ST	POS	130.0867	0.84	$[M+H]^+$	Pipecolic acid	C00408	Lysine degradation	1
ST	POS	124.0395	0.93	$[M+H]^+$	Nicotinic acid	C00253	Nicotinate and nicotinamide metabolism	1
ST	POS	137.0460	1.00	$[M+H]^+$	Hypoxanthine	C00262	Purine metabolism	1
ST	POS	268.1046	1.16	$[M+H]^+$	Adenosine	C00212	Purine metabolism	1
ST	POS	434.1899	1.52	Unknown	UI_12			3
ST	POS	190.0503	3.11	$[M+H]^+$	Kynurenic acid	C01717	Tryptophan metabolism	1
ST	POS	208.0609	3.40	$[M+H]^+$	4-(2-Aminophenyl)-2,4-dioxobutanoic acid	C01252	Tryptophan metabolism	2
ST	POS	269.1755	3.62	Unknown	UI_13			4
ST	POS	192.0660	4.00	Unknown	UI_14			4
ST	POS	174.0553	4.03	Unknown	UI_15			4
ST	POS	197.1177	4.85	Unknown	UI_16			4
ST	POS	105.0702	6.05	Unknown	UI_17			4
ST	POS	379.2963	6.56	Unknown	UI_18			4
ST	POS	299.1286	6.61	$[M+H]^+$	Enterolactone	C18165		1
ST	POS	191.0854	6.69	Unknown	UI_19			4
ST	POS	297.2433	6.89	$[M+H]^+$	Oxylipin (9-HODE)		PUFA derived oxylipins	2
WD	NEG	530.2793	4.79	Unknown	UI_20			4
WD	NEG	385.1439	4.79	Unknown	UI_21			4
WD	NEG	304.6152	5.01	Unknown	UI_22			4
WD	NEG	431.2108	5.14	Unknown	UI_23			4

WD	NEG	512.2682	5.45	$[M-H]^-$	Bile acid_1[6]		Bile acid metabolism	3
WD	NEG	357.1009	5.50	Unknown	UI_24			4
WD	NEG	423.2750	5.71	$[M-H]^-$	Bile acid_2		Bile acid metabolism	4
WD	NEG	469.2806	5.72	$[M-H]^-$	Bile acid_3		Bile acid metabolism	3
WD	NEG	510.2528	5.74	$[M-H_2O-H]^-$	Bile acid_4		Bile acid metabolism	3
WD	NEG	379.2490	5.93	$[M-H]^-$	Bile acid_5		Bile acid metabolism	3
WD	NEG	514.2841	6.09	$[M-H]^-$	Taurocholic acid	C05122	Bile acid metabolism	1
WD	NEG	496.2737	6.10	Unknown	UI_25			4
WD	NEG	487.2367	6.20	$[M-H]^-$	Bile acid_6		Bile acid metabolism	3
WD	NEG	453.2856	6.73	$[M-H]^-$	Bile acid_7		Bile acid metabolism	3
WD	NEG	407.2803	6.74	$[M-H]^-$	Bile acid_8		Bile acid metabolism	3
WD	NEG	405.2645	7.05	$[M-H]^-$	Bile acid_9		Bile acid metabolism	3
WD	NEG	453.2855	7.09	$[M-H]^-$	Bile acid_10		Bile acid metabolism	3
WD	NEG	405.2646	7.58	$[M-H]^-$	Bile acid_11		Bile acid metabolism	3
WD	NEG	451.2701	7.60	$[M-H]^-$	Bile acid_12		Bile acid metabolism	3
WD	NEG	407.2802	7.69	$[M-H]^-$	Cholic acid	C00695	Bile acid metabolism	1
WD	NEG	391.2855	9.31	$[M-H]^-$	Deoxycholic acid	C04483	Bile acid metabolism	1
WD	POS	72.0809	0.85	$[M+H]^+$	Fragment of valine	C00183	Valine, leucine and isoleucine metabolism	1
WD	POS	150.0586	0.98	$[M+H]^+$	Methionine	C00073	Cysteine and methionine metabolism	1
WD	POS	182.0815	1.19	$[M+H]^+$	Tyrosine	C00082	Tyrosine metabolism	1
WD	POS	132.1022	1.31	$[M+H]^+$	Leucine/isoleucine	C00123/C00407	Valine, leucine and isoleucine metabolism	1
WD	POS	137.046	1.35	$[M+H]^+$	Fragment of inosine	C00294	Purine metabolism	1
WD	POS	100.0759	2.11	$[M+H]^+$	2-Piperidone	HMDB0011749		1
WD	POS	166.0865	2.18	$[M+H]^+$	Phenylalanine	C00079	Phenylalanine metabolism	1
WD	POS	188.0710	2.93	$[M+H]^+$	Fragment of tryptophan	C00078	Tryptophan metabolism	1

WD	POS	532.2951	4.78	Unknown	UI_20		4
WD	POS	516.2995	5.38	$[M+H]^+$	Bile acid_13	Bile acid metabolism	3
WD	POS	514.2841	5.45	$[M+H]^+$	Bile acid_14	Bile acid metabolism	3
WD	POS	389.2695	5.71	$[M+H]^+$	Bile acid_15	Bile acid metabolism	3
WD	POS	373.2743	6.37	$[M+H]^+$	Bile acid_16	Bile acid metabolism	3
WD	POS	373.2743	6.73	$[M+H]^+$	Bile acid_17	Bile acid metabolism	3
WD	POS	389.2694	7.06	Unknown	UI_26		4
WD	POS	373.2744	7.11	$[M+H]^+$	Bile acid_18	Bile acid metabolism	3
WD	POS	373.2743	7.69	$[M+H]^+$	Bile acid_19	Bile acid metabolism	3
WD	POS	357.2797	9.30	$[M+H-H_2O]^+$	Bile acid_20	Bile acid metabolism	3
WD	POS	522.3568	10.06	$[M+H]^+$	LysoPC (C26H52NO7P)	Fatty acids and conjugates metabolism	3

[1]Mass spectrometry mass-to-charge ratio; [2]Retention time; [3] KEGG (Kyoto Encyclopedia of Genes and Genomes) compound entry/HMDB (Human Metabolome Data Base) ID; [4]Unidentified; [5]Tentatively identified as a lipid; [6]Tentatively identified as a bile acid

Supplementary Table S4. List of metabolic features discriminating between rats fed WD and WD+high-RE in caecal content

Diet	Ionization mode	m/z[1]	RT[2]	Ion	Metabolite	KEGG/HMDB[3]	Pathway	ID Level
WD	NEG	243.0623	1.09	$[M-H]^-$	Uridine	C00299	Pyrimidine metabolism	2
WD	NEG	385.1439	4.79	Unknown	UI_21[4]			4
WD	NEG	530.2793	4.79	Unknown	UI_20			4
WD	NEG	423.2750	5.71	$[M-H]^-$	Bile acid_2[5]		Bile acid metabolism	3
WD	NEG	469.2806	5.72	$[M-H]^-$	Bile acid_3		Bile acid metabolism	3
WD	NEG	365.2332	5.83	Unknown	UI_27			4
WD	NEG	379.2490	5.93	$[M-H]^-$	Bile acid_5		Bile acid metabolism	3
WD	NEG	421.2595	5.93	$[M-H]^-$	Bile acid_21		Bile acid metabolism	3
WD	NEG	425.2549	5.93	Unknown	UI_28			4
WD	NEG	514.2841	6.09	$[M-H]^-$	Taurocholic acid	C05122	Bile acid metabolism	3
WD	NEG	487.2367	6.20	$[M-H]^-$	Bile acid_6		Bile acid metabolism	3
WD	NEG	423.2750	6.31	$[M-H]^-$	Bile acid_22		Bile acid metabolism	3
WD	NEG	469.2804	6.31	$[M-H]^-$	Bile acid_23		Bile acid metabolism	3
WD	NEG	453.2856	6.73	$[M-H]^-$	Bile acid_24		Bile acid metabolism	3
WD	NEG	407.2803	6.74	$[M-H]^-$	Bile acid_25		Bile acid metabolism	3
WD	NEG	453.2855	7.09	$[M-H]^-$	Bile acid_26		Bile acid metabolism	3
WD	NEG	498.2894	7.12	$[M-H]^-$	Bile acid_27		Bile acid metabolism	3
WD	NEG	405.2646	7.58	$[M-H]^-$	Bile acid_11		Bile acid metabolism	3
WD	NEG	451.2701	7.60	$[M-H]^-$	Bile acid_12		Bile acid metabolism	3
WD	NEG	437.2909	7.88	Unknown	UI_29			4
WD	NEG	391.2854	7.88	$[M-H]^-$	Bile acid_28		Bile acid metabolism	3
WD	NEG	391.2854	8.40	$[M-H]^-$	Bile acid_29		Bile acid metabolism	3

WD	NEG	391.2855	9.31	$[M-H]^-$	Deoxycholic acid	C04483	Bile acid metabolism	1
WD	NEG	437.2910	9.31	$[M-H]^-$	Bile acid_30		Bile acid metabolism	3
WD	POS	286.1046	1.16	$[M+H]^+$	Adenosine	C00212	Purine metabolism	1
WD	POS	188.071	2.93	$[M+H]^+$	Fragment of tryptophan	C00078	Tryptophan metabolism	1
WD	POS	611.345	5.08	Unknown	UI_30			4
WD	POS	516.2995	5.38	$[M+H]^+$	Bile acid_13		Bile acid metabolism	3
WD	POS	407.2801	5.65	$[M+H]^+$	Bile acid_31		Bile acid metabolism	3
WD	POS	389.2695	5.71	$[M+H]^+$	Bile acid_15		Bile acid metabolism	3
WD	POS	405.2643	5.91	$[M+H]^+$	Bile acid_32		Bile acid metabolism	3
WD	POS	345.2431	5.93	$[M+H]^+$	Oxylipin		PUFA derived oxylipins	3
WD	POS	166.0866	6.01	Unknown	UI_31			4
WD	POS	307.2022	6.17	Unknown	UI_32			4
WD	POS	363.2538	6.20	Unknown	UI_33			4
WD	POS	407.28	6.31	$[M+H]^+$	Bile acid_33		Bile acid metabolism	3
WD	POS	373.2743	6.73	$[M+H]^+$	Bile acid_17		Bile acid metabolism	3
WD	POS	373.2744	7.11	$[M+H]^+$	Bile acid_18		Bile acid metabolism	3
WD	POS	389.2695	7.60	$[M+H]^+$	Bile acid_34		Bile acid metabolism	3
WD	POS	407.2802	7.61	$[M+H]^+$	Bile acid_35		Bile acid metabolism	3
WD	POS	357.2796	7.88	Unknown	UI_34			4
WD	POS	357.2797	8.37	Unknown	UI_35			4
WD	POS	391.2853	8.57	$[M+H]^+$	Bile acid_36		Bile acid metabolism	3
WD	POS	375.2903	9.30	$[M+H]^+$	Bile acid_20		Bile acid metabolism	3
WD	POS	785.5949	9.30	$[2M+H]^+$	Bile acid_37		Bile acid metabolism	3
WD+high-RE	NEG	128.0354	1.07	$[M-H]^-$	Pyroglutamic acid	C01879	Glutathione metabolism	1
WD+high-RE	NEG	427.1433	2.94	Unknown	RE_1[6]			4

WD+high-RE	NEG	411.1482	4.12	Unknown	RE_2			4
WD+high-RE	NEG	425.1275	4.16	Unknown	RE_3			4
WD+high-RE	NEG	585.1646	4.63	Unknown	RE_4			4
WD+high-RE	NEG	373.0727	5.13	Unknown	UI_36			4
WD+high-RE	NEG	409.1321	5.30	Unknown	RE_5			4
WD+high-RE	NEG	407.1166	5.31	Unknown	RE_6			4
WD+high-RE	NEG	521.2026	5.60	Unknown	RE_7			4
WD+high-RE	NEG	409.1322	5.95	Unknown	RE_8			4
WD+high-RE	NEG	521.2025	6.16	Unknown	RE_9			4
WD+high-RE	NEG	345.1705	6.92	Unknown	RE_10			4
WD+high-RE	NEG	451.2700	7.06	$[M+FA-H]^-$	Bile acid_38			3
WD+high-RE	NEG	379.1219	7.34	Unknown	RE_11			4
WD+high-RE	NEG	505.2077	7.40	$[M-H]^-$	Carnosol glucoronide			2
WD+high-RE	NEG	345.1705	7.60	Unknown	RE_12			4
WD+high-RE	NEG	507.2235	7.97	$[M-H]^-$	Carnosic acid glucoronide			2
WD+high-RE	NEG	345.1706	8.28	Unknown	RE_13			4
WD+high-RE	NEG	343.1550	9.08	Unknown	RE_14			4
WD+high-RE	NEG	329.1757	9.46	$[M-H]^-$	Carnosol	C09069		1
WD+high-RE	NEG	285.1861	9.46	Unknown	RE_15			4
WD+high-RE	NEG	331.1911	10.50	$[M-H]^-$	Carnosic acid	C21818		1
WD+high-RE	NEG	329.1758	10.50	Unknown	RE_16			4
WD+high-RE	NEG	317.2123	11.54	Unknown	RE_17			4
WD+high-RE	POS	101.0599	0.77	Unknown	UI_37			4
WD+high-RE	POS	118.0865	0.78	$[M+H]^+$	Betaine	C00719	Glycine, serine and threonine metabolism	1
WD+high-RE	POS	254.1617	1.61	Unknown	UI_38			4

WD+high-RE	POS	450.1953	4.63	Unknown	RE_18		4
WD+high-RE	POS	411.1480	5.28	Unknown	RE_19		4
WD+high-RE	POS	450.1953	5.39	Unknown	RE_20		4
WD+high-RE	POS	329.1754	5.40	Unknown	RE_21		4
WD+high-RE	POS	636.2597	5.52	Unknown	RE_22		4
WD+high-RE	POS	432.2754	5.60	Unknown	RE_23		4
WD+high-RE	POS	450.1952	5.65	Unknown	RE_24		4
WD+high-RE	POS	452.2109	5.84	Unknown	RE_25		4
WD+high-RE	POS	329.1755	5.93	Unknown	RE_26		4
WD+high-RE	POS	638.1750	6.01	Unknown	RE_27		4
WD+high-RE	POS	432.2752	6.03	Unknown	RE_28		4
WD+high-RE	POS	448.1797	6.10	Unknown	RE_29		4
WD+high-RE	POS	347.1860	6.18	Unknown	RE_30		4
WD+high-RE	POS	329.1754	6.93	Unknown	RE_31		4
WD+high-RE	POS	461.2180	7.40	Unknown	RE_32		4
WD+high-RE	POS	331.1911	7.41	Unknown	RE_33		4
WD+high-RE	POS	301.1805	7.59	Unknown	RE_34		4
WD+high-RE	POS	329.1755	7.59	Unknown	RE_35		4
WD+high-RE	POS	373.2743	7.69	Unknown	RE_36		4
WD+high-RE	POS	463.2335	7.96	Unknown	RE_37		4
WD+high-RE	POS	329.1754	8.24	Unknown	RE_38		4
WD+high-RE	POS	331.1912	9.45	$[M+H]^+$	Carnosol	C09069	1
WD+high-RE	POS	299.0922	9.68	Unknown	RE_39		4
WD+high-RE	POS	287.2013	10.49	$[M+H]^+$	Fragment of Carnosic acid	C21818	1

[1]Mass spectrometry mass-to-charge ratio; [2]Retention time; [3] KEGG (Kyoto Encyclopedia of Genes and Genomes) compound entry/HMDB (Human Metabolome Data Base) ID; [4]Unidentified; [5]Tentatively identified as a bile acid; [6]Tentatively identified as a metabolite of rosemary extract.

Supplementary Table S5. List of metabolic features discriminating between rats fed ST and WD in plasma

Diet	Ionization mode	m/z[1]	RT[2]	Ion	Metabolite	KEGG/HMDB[3]	Pathway	ID Level
ST	NEG	103.0401	1.31	Unknown	UI_1[4]			4
ST	NEG	188.9864	3.02	$[M-H]^-$	Pyrocatechol sulfate	HMDB0059724	Potential urinary biomarker of whole grain intake	2
ST	NEG	172.9915	3.27	$[M-H]^-$	Phenol sulfate	C02180		2
ST	NEG	178.0512	3.62	$[M-H]^-$	Hippuric acid	C01586	Phenylalanine metabolism	1
ST	NEG	212.0024	3.66	$[M-H]^-$	Indoxyl sulfate	HMDB0000682	Tryptophan metabolism	1
ST	NEG	165.0559	4.44	$[M-H]^-$	D-Phenyllactic acid	C05607	Phenylalanine metabolism	1
ST	NEG	417.1192	4.80	$[M-H]^-$	Equol 7-O-glucuronide	HMDB0041732	Polyphenol metabolite	2
ST	NEG	201.0229	5.13	Unknown	UI_2			4
ST	NEG	202.0260	5.13	Unknown	UI_3			4
ST	NEG	343.0859	5.28	Unknown	UI_4			4
ST	NEG	514.2845	5.38	$[M-H]^-$	BA_1[5]		Bile acid metabolism	3
ST	NEG	514.2848	6.09	$[M-H]^-$	BA_2		Bile acid metabolism	3
ST	NEG	498.2896	6.88	$[M-H]^-$	BA_3		Bile acid metabolism	3
ST	NEG	453.2860	7.07	$[M-H]^-$	BA_4		Bile acid metabolism	3
ST	NEG	407.2805	7.07	$[M-H]^-$	BA_5		Bile acid metabolism	3
ST	NEG	407.2806	7.67	$[M-H]^-$	Cholic acid	C00695	Bile acid metabolism	1
ST	NEG	437.2912	7.86	Unknown	UI_5			4
ST	NEG	437.2914	9.09	$[M+FA-H]^-$	BA_6		Bile acid metabolism	
ST	NEG	554.3470	10.36	$[M+FA-H]^-$	PC(17:0/0:0)[6]			3
ST	POS	118.0866	0.72	$[M+H]^+$	Betaine	C00719	Glycine, serine and threonine metabolism	1
ST	POS	162.1129	0.73	$[M+H]^+$	Carnitine	C00487	Lysine degradation	1
ST	POS	90.0552	0.74	$[M+H]^+$	Alanine/Sarcosine	C00041/C00213	Amino acid metabolism	1

ST	POS	132.077	0.75	$[M+H]^+$	Creatine	C00300	Glycine, serine and threonine metabolism	1
ST	POS	138.0544	0.75	$[M+H]^+$	N-Methylnicotinate	C01004	Nicotinate and nicotinamide metabolism	2
ST	POS	144.1022	0.77	$[M+H]^+$	Proline betaine	C10172		2
ST	POS	158.1179	0.87	$[M+H]^+$	Pipecolic acid betaine	C08283		2
ST	POS	204.1235	0.94	$[M+H]^+$	Acetylcarnitine	C02571		1
ST	POS	276.1448	1.25	$[M+H]^+$	Glutarylcarnitine	HMDB0013130		2
ST	POS	294.1554	1.25	$[M+H]^+$	N-(1-Deoxy-1-fructosyl)leucine	HMDB0037840		2
ST	POS	218.1392	1.52	$[M+H]^+$	Propionylcarnitine	C03017		1
ST	POS	331.1329	2.59	Unknown	UI_6			4
ST	POS	232.1549	2.60	$[M+H]^+$	Butyrylcarnitine	C02862		1
ST	POS	105.0338	3.62	Unknown	UI_7			4
ST	POS	180.0660	3.62	$[M+H]^+$	Hippuric acid	C01586	Phenylalanine metabolism	1
ST	POS	190.0867	5.99	$[M+H]^+$	3-Indolepropionic acid	C11284	Tryptophan metabolism	1
ST	POS	373.2746	7.67	$[M+H]^+$	Cholic acid	C00695	Bile acid metabolism	1
ST	POS	357.2797	7.86	Unknown	UI_8			3
ST	POS	299.2014	8.24	Unknown	UI_9			4
ST	POS	357.2796	9.10	Unknown	UI_10			3
ST	POS	570.3565	9.60	$[M+H]^+$	PC(22:5/0:0)		Fatty acids and conjugates metabolism	3
ST	POS	496.3407	9.74	$[M+H]^+$	PC(0:0/16:0)		Fatty acids and conjugates metabolism	3
ST	POS	510.3567	10.35	$[M+H]^+$	PC(17:0/0:0)		Fatty acids and conjugates metabolism	3
WD	NEG	133.0143	0.89	$[M-H]^-$	Malic acid	C00149	Citrate cycle (TCA cycle)	1
WD	NEG	291.0833	0.92	Unknown	UI_11			4
WD	NEG	129.0559	3.31	$[M-H]^-$	3-Methyl-2-oxovaleric acid	HMDB0000491	Valine, leucine and isoleucine degradation	1
WD	NEG	129.0559	3.64	$[M-H]^-$	4-Methyl-2-oxovaleric acid	C00233	Valine, leucine and isoleucine degradation	1
WD	NEG	187.0072	4.23	$[M-H]^-$	p-cresol sulfate	HMDB0011635	Amino acid metabolism	1

WD	NEG	512.2999	8.55	$[M+FA-H]^-$	PC(14:0/0:0)		Fatty acids and conjugates metabolism	2
WD	NEG	586.3155	8.78	$[M+FA-H]^-$	PC(20:5/0:0)		Fatty acids and conjugates metabolism	2
WD	NEG	538.3156	8.92	$[M+FA-H]^-$	PC(16:1/0:0)		Fatty acids and conjugates metabolism	2
WD	NEG	526.3156	9.12	$[M+FA-H]^-$	PC(15:0/0:0)		Fatty acids and conjugates metabolism	2
WD	NEG	500.2789	9.29	$[M-H]^-$	PE(20:4/0:0)[7]		Fatty acids and conjugates metabolism	2
WD	NEG	564.3313	9.31	$[M+FA-H]^-$	PC(18:2/0:0)		Fatty acids and conjugates metabolism	2
WD	NEG	612.3311	9.31	$[M+FA-H]^-$	PC(22:6/0:0)		Fatty acids and conjugates metabolism	3
WD	NEG	588.3312	9.35	$[M+FA-H]^-$	PC(20:4/0:0)		Fatty acids and conjugates metabolism	2
WD	NEG	552.3311	9.47	$[M+FA-H]^-$	PC(17:1/0:0)		Fatty acids and conjugates metabolism	2
WD	NEG	590.3470	9.76	$[M+FA-H]^-$	PC(20:3/0:0)		Fatty acids and conjugates metabolism	3
WD	NEG	478.2944	9.98	$[M-H]^-$	PE(18:1/0:0)		Fatty acids and conjugates metabolism	2
WD	NEG	566.3469	10.05	$[M+FA-H]^-$	PC(18:1/0:0)		Fatty acids and conjugates metabolism	2
WD	NEG	638.2770	10.05	Unknown	UI_12			4
WD	NEG	634.3342	10.05	Unknown	UI_13			4
WD	NEG	568.3624	11.02	$[M+FA-H]^-$	PC(18:0/0:0)		Fatty acids and conjugates metabolism	2
WD	POS	145.0499	0.72	Unknown	UI_14			4
WD	POS	116.0709	0.76	$[M+H]^+$	Proline	C00148	Arginine and proline metabolism	1
WD	POS	455.1893	0.84	Unknown	UI_15			4
WD	POS	123.0556	0.94	$[M+H]^+$	Niacinamide	C00153	Nicotinate and nicotinamide metabolism	1
WD	POS	100.0760	2.12	$[M+H]^+$	δ-Valerolactam	C02240		1
WD	POS	188.0710	2.93	$[M+H]^+$	Tryptophan	C00078	Tryptophan metabolism	1
WD	POS	468.3097	8.55	$[M+H]^+$	PC(14:0/0:0)		Fatty acids and conjugates metabolism	2
WD	POS	542.3254	8.77	$[M+H]^+$	PC(20:5/0:0)		Fatty acids and conjugates metabolism	2
WD	POS	518.3252	8.82	$[M+H]^+$	PC(18:3)		Fatty acids and conjugates metabolism	2
WD	POS	494.3253	8.91	$[M+H]^+$	PC(16:1/0:0)		Fatty acids and conjugates metabolism	2

WD	POS	516.3070	8.91	Unknown	UI_16		4
WD	POS	482.3253	9.12	$[M+H]^+$	PC(15:0/0:0)	Fatty acids and conjugates metabolism	2
WD	POS	502.3929	9.29	$[M+H-H2O]^+$	PC(18:2/0:0)	Fatty acids and conjugates metabolism	2
WD	POS	568.3407	9.31	$[M+H]^+$	PC(22:6/0:0)	Fatty acids and conjugates metabolism	2
WD	POS	544.3408	9.34	$[M+H]^+$	PC(20:4/0:0)	Fatty acids and conjugates metabolism	2
WD	POS	508.3408	9.48	$[M+H]^+$	PC(17:1/0:0)	Fatty acids and conjugates metabolism	2
WD	POS	546.3565	9.77	$[M+H]^+$	PC(20:3/0:0)	Fatty acids and conjugates metabolism	2
WD	POS	426.3588	9.87	Unknown	UI_17		4
WD	POS	570.3566	9.92	$[M+H]^+$	PC(22:5/0:0)	Fatty acids and conjugates metabolism	3
WD	POS	480.3095	9.99	$[M+H]^+$	PE(18:1/0:0)	Fatty acids and conjugates metabolism	3
WD	POS	522.3567	10.06	$[M+H]^+$	PC(18:1/0:0)	Fatty acids and conjugates metabolism	2
WD	POS	289.6428	10.06	Unknown	UI_18		4
WD	POS	550.3165	10.06	$[M+H]^+$	PS(20:2/0:0)[8]	Fatty acids and conjugates metabolism	3
WD	POS	510.3567	10.15	$[M+H]^+$	PC/PE (C25H52NO7P)	Fatty acids and conjugates metabolism	3
WD	POS	536.3721	10.65	$[M+H]^+$	PC/PE (C27H54NO7P)	Fatty acids and conjugates metabolism	3
WD	POS	524.3725	11.02	$[M+H]^+$	PC(18:0/0:0)	Fatty acids and conjugates metabolism	3

[1]Mass spectrometry mass-to-charge ratio; [2]Retention time; [3] KEGG (Kyoto Encyclopedia of Genes and Genomes) compound entry/HMDB (Human Metabolome Data Base) ID; [4]Unidentified; [5]Tentatively identified as a bile acid; [6]Tentatively identified as a phosphatidylcholine; [7]Tentatively identified as a phosphatidylethanolamine; [8]Tentatively identified as a phosphatidylserine.

Supplementary Table S6. List of metabolic features discriminating between rats fed WD and WD+high-RE in plasma.

Diet	Ionization mode	m/z[1]	RT[2]	Ion	Metabolite	KEGG/HMDB[3]	Pathway	ID Level
WD	NEG	215.0328	0.72	$[M+Cl]^-$	C6H12O6, Cl-adduct			3
WD	NEG	167.0211	1.00	$[M-H]^-$	Uric acid	C00366	Purine metabolism	1
WD	NEG	103.0401	1.62	Unknown	UI_19[4]			4
WD	NEG	129.0559	3.31	$[M-H]^-$	3-Methyl-2-oxovaleric acid	HMDB0000491	Valine, leucine and isoleucine degradation	1
WD	NEG	129.0559	3.64	$[M-H]^-$	4-Methyl-2-oxovaleric acid	C00233	Valine, leucine and isoleucine degradation	1
WD	NEG	187.0072	4.23	$[M-H]^-$	p-cresol sulfate	HMDB0011635	Amino acid metabolism	1
WD	NEG	357.1014	5.52	Unknown	UI_20			4
WD	NEG	538.3156	8.92	$[M+FA-H]^-$	PC(16:1/0:0)[5]		Fatty acids and conjugates metabolism	3
WD	NEG	564.3313	9.31	$[M+FA-H]^-$	PC(18:2/0:0)		Fatty acids and conjugates metabolism	3
WD	NEG	612.3311	9.31	$[M+FA-H]^-$	PC(22:6/0:0)		Fatty acids and conjugates metabolism	3
WD	NEG	588.3312	9.35	$[M+FA-H]^-$	PC(20:4/0:0)		Fatty acids and conjugates metabolism	3
WD	NEG	540.3313	9.45	$[M+FA-H]^-$	PC(16:0/0:0)		Fatty acids and conjugates metabolism	3
WD	NEG	540.3313	9.72	$[M+FA-H]^-$	PC(16:0/0:0)		Fatty acids and conjugates metabolism	3
WD	NEG	590.3470	9.76	$[M+FA-H]^-$	PC(20:3/0:0)		Fatty acids and conjugates metabolism	3
WD	NEG	566.3469	10.05	$[M+FA-H]^-$	PC(18:1/0:0)		Fatty acids and conjugates metabolism	3
WD	NEG	554.3470	10.36	$[M+FA-H]^-$	PC(17:0/0:0)		Fatty acids and conjugates metabolism	3
WD	NEG	568.3625	10.74	$[M+FA-H]^-$	PC(18:0/0:0)		Fatty acids and conjugates metabolism	3
WD	NEG	480.3100	10.96	$[M-H]^-$	PE(18:0/0:0)[6]		Fatty acids and conjugates metabolism	3
WD	NEG	568.3624	11.02	$[M+FA-H]^-$	PC(18:0/0:0)		Fatty acids and conjugates metabolism	3
WD	POS	203.0530	0.73	$[M+Na]^+$	C6H12O6, Na-adduct			3
WD	POS	169.0360	0.94	$[M+H]^+$	Uric acid	C00366	Purine metabolism	1
WD	POS	153.0662	1.39	Unknown	UI_21			4

WD	POS	100.0760	2.12	$[M+H]^+$	δ-Valerolactam	C02240		1
WD	POS	166.0867	2.18	$[M+H]^+$	Phenylalanine	C00079	Phenylalanine metabolism	1
WD	POS	373.2752	6.73	$[M+H]^+$	BA_7[7]		Bile acid metabolism	3
WD	POS	494.3253	8.91	$[M+H]^+$	PC(16:1/0:0)		Fatty acids and conjugates metabolism	2
WD	POS	357.2796	9.10	Unknown	Lipid or bile acid			3
WD	POS	520.3408	9.31	$[M+H]^+$	PC(18:2/0:0)		Fatty acids and conjugates metabolism	3
WD	POS	544.3408	9.34	$[M+H]^+$	PC(20:4/0:0)		Fatty acids and conjugates metabolism	3
WD	POS	570.3565	9.60	$[M+H]^+$	PC(22:5/0:0)		Fatty acids and conjugates metabolism	3
WD	POS	496.3407	9.74	$[M+H]^+$	PC(0:0/16:0)		Fatty acids and conjugates metabolism	3
WD	POS	546.3565	9.77	$[M+H]^+$	PC(20:3/0:0)		Fatty acids and conjugates metabolism	3
WD	POS	570.3566	9.92	$[M+H]^+$	PC(22:5/0:0)		Fatty acids and conjugates metabolism	3
WD	POS	522.3567	10.06	$[M+H]^+$	PC(18:1/0:0)		Fatty acids and conjugates metabolism	3
WD	POS	510.3567	10.15	$[M+H]^+$	PC/PE (C25H52NO7P)		Fatty acids and conjugates metabolism	3
WD	POS	524.3724	10.73	$[M+H]^+$	PC(18:0/0:0)		Fatty acids and conjugates metabolism	3
WD	POS	524.3725	11.02	$[M+H]^+$	PC(18:0/0:0)		Fatty acids and conjugates metabolism	3
WD+high-RE	NEG	313.0652	0.82	Unknown	RE_1[8]			4
WD+high-RE	NEG	329.0391	0.82	Unknown	RE_2			4
WD+high-RE	NEG	191.0197	1.08	$[M-H]^-$	Citric acid	C00158	Citrate cycle (TCA cycle)	1
WD+high-RE	NEG	172.9915	3.27	$[M-H]^-$	Phenyl sulfate	HMDB0060015		2
WD+high-RE	NEG	505.2084	7.31	Unknown	RE_3			4
WD+high-RE	NEG	507.2240	7.95	Unknown	RE_4			4
WD+high-RE	NEG	317.1761	8.00	Unknown	RE_5			4
WD+high-RE	NEG	361.1659	8.00	Unknown	RE_6			4
WD+high-RE	NEG	301.1810	8.21	Unknown	RE_7			4
WD+high-RE	NEG	346.1743	8.22	Unknown	RE_8			4

WD+high-RE	NEG	345.1710	8.23	$[M-H]^-$	Rosmanol/Epiisorosmanol			3
WD+high-RE	NEG	359.1504	8.62	Unknown	RE_9			4
WD+high-RE	NEG	329.1760	9.45	$[M-H]^-$	Carnosol	C09069		1
WD+high-RE	NEG	359.1504	9.98	Unknown	RE_10			4
WD+high-RE	NEG	343.1554	10.02	$[M-H]^-$	Rosmadial	HMDB0038219		2
WD+high-RE	NEG	299.1653	10.23	Unknown	RE_11			4
WD+high-RE	NEG	345.1710	10.24	$[M-H]^-$	Rosmanol/Epiisorosmanol			3
WD+high-RE	NEG	331.1917	10.48	$[M-H]^-$	Carnosic acid	C21818		1
WD+high-RE	NEG	332.1952	10.48	Unknown	RE_12			4
WD+high-RE	NEG	345.2073	11.05	$[M-H]^-$	Methyl carnosate			2
WD+high-RE	POS	118.0866	0.73	$[M+H]^+$	Betaine	C00719	Glycine, serine and threonine metabolism	1
WD+high-RE	POS	132.0770	0.75	$[M+H]^+$	Creatine	C00300	Amino acid metabolism	1
WD+high-RE	POS	130.0502	1.06	$[M+H]^+$	Pyroglutamic acid	C01879	Glutathione metabolism	1
WD+high-RE	POS	160.0760	1.91	Unknown	UI_22			4
WD+high-RE	POS	188.0710	2.93	$[M+H]^+$	Tryptophan	C00078	Tryptophan metabolism	1
WD+high-RE	POS	318.2073	5.78	Unknown	RE_13			4
WD+high-RE	POS	277.1396	6.37	Unknown	UI_23			4
WD+high-RE	POS	318.2073	6.38	Unknown	RE_14			4
WD+high-RE	POS	463.2337	7.95	Unknown	RE_15			4
WD+high-RE	POS	302.2124	7.98	Unknown	RE_16			4
WD+high-RE	POS	363.1811	8.02	Unknown	RE_6			4
WD+high-RE	POS	369.1683	8.21	Unknown	RE_17			4
WD+high-RE	POS	347.1862	8.22	Unknown	RE_18			4
WD+high-RE	POS	316.1918	8.53	Unknown	RE_19			4
WD+high-RE	POS	331.1913	9.44	$[M+H]^+$	Carnosol	C09069		1

WD+high-RE	POS	302.2123	10.22	Unknown	RE_20		4
WD+high-RE	POS	347.1860	10.23	Unknown	RE_21		4
WD+high-RE	POS	301.1804	10.23	Unknown	RE_12		4
WD+high-RE	POS	287.2013	10.47	$[M+H]^+$	Carnosic acid frag	C21818	1
WD+high-RE	POS	301.2170	11.06	Unknown	RE_22		4

[1]Mass spectrometry mass-to-charge ratio; [2]Retention time; [3] KEGG (Kyoto Encyclopedia of Genes and Genomes) compound entry/HMDB (Human Metabolome Data Base) ID; [4]Unidentified; [5]Tentatively identified as a phosphatidylcholine; [6]Tentatively identified as a phosphatidylethanolamine; [7]Tentatively identified as a bile acid; [8]Tentatively identified as a metabolite of rosemary extract.
